# 30-day in-hospital stroke case fatality and significant risk factors in sub-Saharan–Africa: A systematic review and meta-analysis

**DOI:** 10.1371/journal.pgph.0002769

**Published:** 2024-01-19

**Authors:** Martin Ackah, Louise Ameyaw, Richard Appiah, David Owiredu, Hosea Boakye, Webster Donaldy, Comos Yarfi, Ulric S. Abonie

**Affiliations:** 1 Faculty of Health and Life Sciences Northumbria University University, Department of Sport, Exercise & Rehabilitation, Newcastle upon Tyne, United Kingdom; 2 Department of Medicine, Achimota Government Hospital, Accra, Ghana; 3 Faculty of Health and Life Sciences Northumbria University University, Department of Psychology, Newcastle upon Tyne, United Kingdom; 4 Department of Occupational therapy, College of Health Sciences, University of Ghana, Korle-Bu, Accra, Ghana; 5 Centre for Evidence synthesis, University of Ghana, Accra, Ghana; 6 Department of Physiotherapy, LEKMA Hospital, Accra, Ghana; 7 Harlem Hospital Center, New York, United States of America; 8 Department of Physiotherapy, University of Allied and Health Sciences, Ho, Ghana; Kwame Nkrumah University of Science and Technology, GHANA

## Abstract

Existing studies investigating 30-day in-hospital stroke case fatality rates in sub-Saharan Africa have produced varying results, underscoring the significance of obtaining precise and reliable estimations for this indicator. Consequently, this study aimed to conduct a systematic review and update of the current scientific evidence regarding 30-day in-hospital stroke case fatality and associated risk factors in sub-Saharan Africa. Medline/PubMed, Cumulative Index to Nursing and Allied Health Literature (CINAHL), APA PsycNet (encompassing PsycINFO and PsychArticle), Google Scholar, and Africa Journal Online (AJOL) were systematically searched to identify potentially relevant articles. Two independent assessors extracted the data from the eligible studies using a pre-tested and standardized excel spreadsheet. Outcomes were 30-day in-hospital stroke case fatality and associated risk factors. Data was pooled using random effects model. Ninety-three (93) studies involving 42,057 participants were included. The overall stroke case fatality rate was 27% [25%-29%]. Subgroup analysis revealed 24% [21%-28%], 25% [21%-28%], 29% [25%-32%] and 31% [20%-43%] stroke case fatality rates in East Africa, Southern Africa, West Africa, and Central Africa respectively. Stroke severity, stroke type, untyped stroke, and post-stroke complications were identified as risk factors. The most prevalent risk factors were low (<8) Glasgow Coma Scale score, high (≥10) National Institute Health Stroke Scale score, aspiration pneumonia, hemorrhagic stroke, brain edema/intra-cranial pressure, hyperglycemia, untyped stroke (stroke diagnosis not confirmed by neuroimaging), recurrent stroke and fever. The findings indicate that one in every four in-hospital people with stroke in sub-Saharan Africa dies within 30 days of admission. Importantly, the identified risk factors are mostly modifiable and preventable, highlighting the need for context-driven health policies, clinical guidelines, and treatments targeting these factors.

## Introduction

Stroke is the second most significant contributor to global mortality and the third most prevalent cause of combined death and disability, accounting for 12% of overall deaths and 6% of combined death and disability [1,2]. The incidence of stroke in Africa continues to increase and is among the highest in the world (2), with an age-adjusted incidence rate of 316 per 100,000 people and a prevalence of 1460 per 100,000 people per year [3–5]. It is estimated that nine out of ten burden of stroke is attributable to modifiable risk factors with regional variations [6]. In the sub-Saharan Africa (SSA) region, hypertension, dyslipidemia, regular meat eating, an elevated waist-to-hip ratio, diabetes, a low intake of green leafy vegetables, stress, table salt, heart disease, physical inactivity and tobacco use have been identified as leading risk factors associated with stroke [7–9].

In-hospital stroke mortality rates in SSA vary significantly, ranging from 1.5% to 77.8% [10,11]. A systematic review and meta-analysis found that 22% of the overall prevalence of in-hospital mortality in SSA was linked to stroke [12]. However, a considerable number of cohort studies have been published after this review, necessitating a review to comprehensively identify risk factors and capture current estimates of in-hospital stroke mortality in SSA. Thirty-day in-hospital stroke mortality refers to the percentage of people who die within 30 days of being admitted to the hospital for stroke [13]. The 30-day mortality rate following hospital admission is a commonly utilized metric to evaluate hospital performance [14]. The choice of this standardized timeframe was to ensure an equitable evaluation, mitigating the impact of transfer rates or variations in length of stay on the measurement [14,15].

Generally, acute medical and rehabilitation care for stroke survivors is limited and underfunded in SSA [16]. Cost-effective and pragmatic preventative programs aimed at identifying and managing risk factors are thus critical in reducing the burden of stroke in SSA [7]. Such efforts could also improve the survival rate of in-patients with high risk of mortality through timely intervention and care [17]. Consequently, evaluation of 30-day in-hospital stroke case fatality and associated risk factors in SSA may be an important step to reduce case fatality in this population [13]. This is essential to help guide policy formulation and treatment effort to reduce stroke-related case fatality. Therefore, the present study sought to systematically review and update scientific evidence on the 30-day case fatality and associated risk factors among in-hospital persons with stroke in SSA.

## Methods

### Protocol registration and best practices

The systematic review and meta-analysis were conducted in accordance with the guidelines outlined in the Preferred Reporting Items for Systematic Review and Meta-Analysis (PRISMA) [18] to ensure the application of best practices. The review was registered with the International Prospective Register of Systematic Reviews (PROSPERO) database (registration number: CRD42021227367). The protocol for this review has been published elsewhere [13].

### Eligibility criteria

Studies reporting 30-day in-hospital stroke case-fatality and/or associated risk factors in any SSA country were eligible for inclusion. Persons diagnosed with stroke and on admission in any health facility in SSA, irrespective of age, were included. Reviews, commentaries, letters of correspondence, community studies, systematic reviews and conference papers were excluded.

The primary and secondary outcomes were in-hospital 30-day stroke case fatality rate and risk factors associated with in-hospital 30-day stroke case fatality respectively.

### Search methods for identification of studies

Medline (via PubMed), Cumulative Index to Nursing and Allied Health Literature (CINAHL), APA PsycNet (encompassing PsycINFO and PsychArticle), Google Scholar, and African Journals Online (AJOL) were searched for publications on the rate and risk factors of in-hospital stroke mortality/case-fatality in SSA. The search was limited to papers published in English from January 1990 to September 2023. The full search strategy is presented in S1 Table. Hand searches of the reference lists of relevant articles were conducted to identify additional eligible studies.

### Screening, selection of studies, and data extraction

All search results were collated and deduplicated using Mendeley reference manager software. Two authors independently (MA and DO) screened the titles and abstracts of all studies against the pre-specified eligibility criteria using a pretested study selection chart. The screening of titles and abstracts was conducted manually. Full texts of all potentially eligible studies were accessed through PubMed, Google Scholar, and the respective websites of individual journals such as Elsevier, Lancet, and Sage, and further assessed for eligibility. Disagreements on inclusion decisions were resolved through discussion between the two reviewers or by consulting a third independent reviewer (USA). Corresponding authors of studies whose full texts were not accessible were contacted through email to provide them. If the full text was still inaccessible and vital information needed to decide on eligibility and inclusion was unavailable the study was excluded. The PRISMA flow chart was used to summarize the study selection process. Two independent reviewers (MA and DO) pilot-tested the data extraction template (a Microsoft Excel sheet) with 10% of the included studies, before commencing data extraction for all the included studies. Collated data included author’s last name, year of publication, study country, participants’ age, sample size, 30-day in-hospital stroke case fatality and associated risk factors.

### Outcome and operationalization

30-day in-hospital stroke case fatality rate was defined as the proportion of persons with stroke who died within 30 days of hospital admission. A risk factor was also defined as a variable that was linked to, or caused the death of, a hospitalized person with stroke within 30 days of admission.

### Risk of bias and quality assessment

The methodological quality of the included studies was independently assessed by two reviewers (MA and USA) using a 10-item tool for assessing risk of bias in prevalence studies [19]. The tool assesses characteristics of reporting internal and external validity of studies. Risk of bias (low = 7–10; moderate = 4–6; high = 0–3) was classified based on the following items from this tool: national and target population representativeness, sampling frame representativeness, selection method, response bias, data quality, case definition, reliability and validity of study instrument, data collection consistency, prevalence duration, and parameter suitability. Discrepancies in quality and risk rating were resolved by consensus between the two reviewers.

### Data synthesis

Extracted data were exported to Stata (version 16; Stata Corp, TX, USA) from Microsoft excel 2013 for statistical analyses. The Clopper-Pearson method [i.e., ‘cimethod (exact)’] was used to determine the study-specific confidence intervals [20], to ensure that the resulting intervals always contain valid and permissible values [21]. The rate of 30-day in-hospital stroke case fatality was pooled using a random-effects model. Heterogeneity was visually inspected using the forest plot and quantified using both Cochrane’s Q statistic and the I^2^ statistic [22]. The I^2^ values were interpreted based on the Higgins and Thompson classification, with 25%, 50% and 75% reflecting low, moderate and high heterogeneity, respectively [22]. Sub-group analysis was conducted (where applicable) to examine whether estimates varied according to the potential moderators such as sub-region (West Africa, East Africa, Southern Africa, and Central Africa). A risk factor was eligible for inclusion if it had been adjusted and reported in included studies [23].

## Results

### Study selection

**Fig 1:**
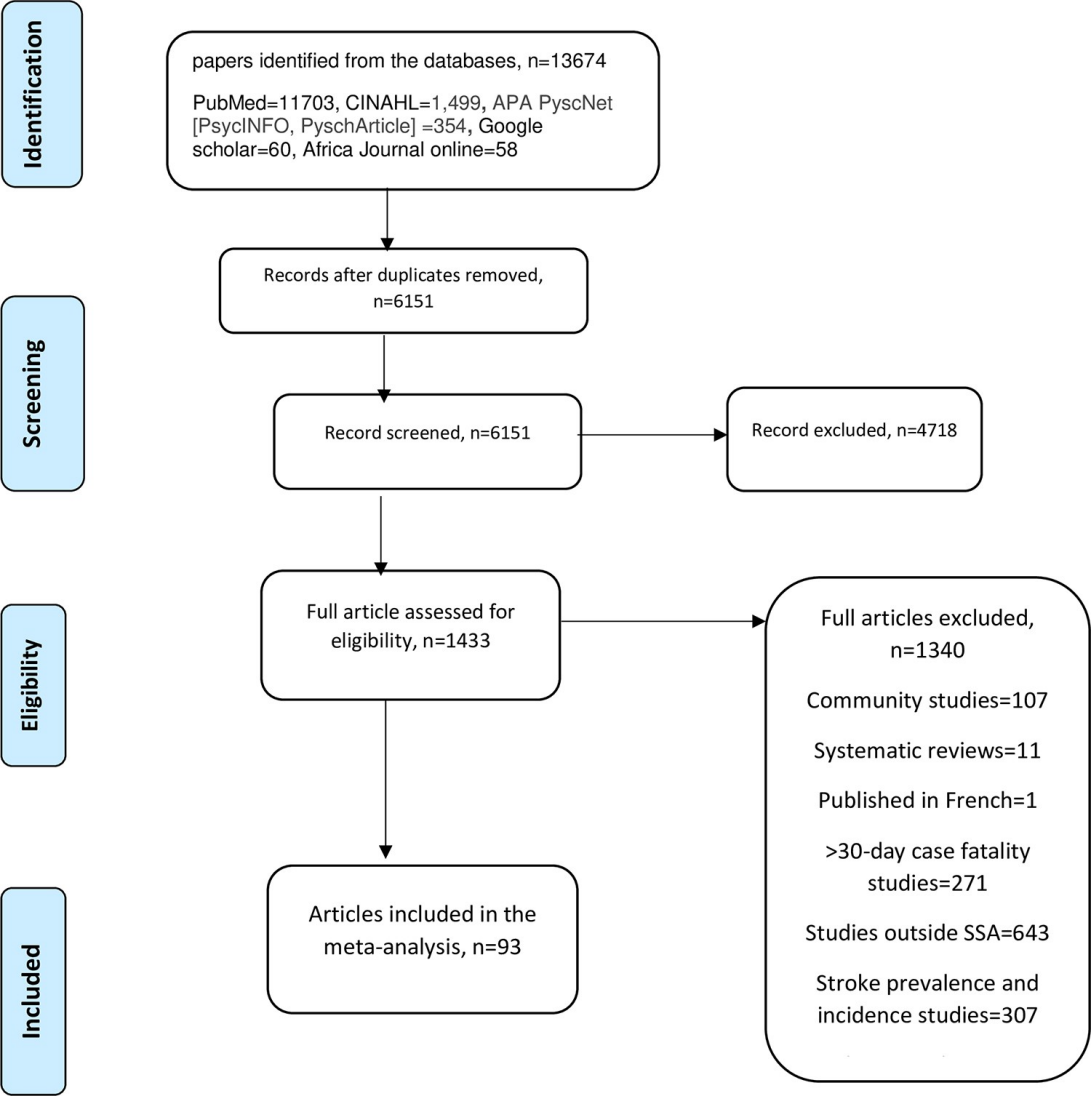
Flow chart of study selection, adapted from PRISMA.

Fig 1 shows the data identification process. A total of 13674 potentially relevant studies were identified in literature search and additional hand searches. After removal of duplicates, the abstract of 6151 studies were screened for eligibility based on the inclusion criteria. After screening of abstracts, 4718 studies were excluded. Common reasons for exclusion were inappropriate outcome measures, inappropriate study designs and inappropriate sample groups. A total of 1433 full-text articles were retrieved. Six hundred and forty-three studies were conducted outside SSA, three hundred and seven studies were stroke prevalence and incidence studies, two hundred and seventy-one studies reported over 30-day case fatality, a hundred and seven studies were community studies, eleven studies were reviews, and one study was published in French, and hence were also excluded. This resulted in ninety-three studies [11,24–115] eligible for inclusion.

### Study characteristics

The characteristics of the included studies are presented in Table 1. A total of 42,057 participants were involved in the studies. Studies were published between 2002 and 2023. The sample size ranged from 35 to 12,233 participants. Participants’ ages ranged from 16 to 115 years. Most of the studies were conducted in West Africa (43 out of 93 studies), followed by East Africa (42 out of 93 studies). In terms of country, most of the studies were conducted in Nigeria (24 out of 93 studies) and Ethiopia (22 out of 93 studies).

**Table 1:** Characteristics of the included studies.

Study	Year of Publication	Country	Participants Age [years]	Sample Size	30-day Case fatality [%]
Alemayehu, et al., [11]	2002	Ethiopia		259	1.5
Namale et al., [24]	2020	Uganda		141	22.0
Mulugeta et al., [25]	2020	Ethiopia	18–65+	162	8.6
Gadisa, et al., [26]	2020	Ethiopia	63.4±12.6	111	16.2
Dabilgou, et al., [27]	2020	Burkina Faso	26–90	302	39.1
Fekadu, et al., [28]	2020	Ethiopia		116	29.3
Kamabu, et al., [29]	2020	Dr. Congo		180	31.7
Lisk, et al.,[30]	2020	Sierra Leone		170	9.4
Adoukonou et al., [31]	2020	Benin	58.2±14.2	372	6.2
Kayode-Iyasere et al., [32]	2019	Nigeria	18–99	419	37.2
Gebreyohannes, et al., [33]	2019	Ethiopia	65.2±14.1	208	12.5
Fekadu, et al., [34]	2019	Ethiopia	23–96	116	21.6
Tshituta et al., [35]	2019	DR Congo	58.7 ± 13.1	194	48.5
Erkabu, et al., [36]	2018	Ethiopia	18–70+	303	10.6
Asefa, et al., [37]	2018	Ethiopia	25–65+	367	37.9
Kaduka, et al., [38]	2018	Kenya		719	26.7
Mbelesso et al., [39]	2018	Central Africa Republic	38–91	154	29,9
Labodi et al., [40]	2017	Burkina Faso	28–92	157	28.7
Gedefa et al., [41]	2017	Ethiopia	25–85+	163	30.1
Okeng’o et al., [42]	2017	Tanzania	20–104	186	33.3
Kaseke et al., [43]	2017	Zimbabwe		450	24.0
Sultan et al., [44]	2017	Ethiopia		301	19.3
Owolabi et al., [45]	2016	Nigeria	38–80	536	15.9
Lekoubou, et al., [46]	2015	Cameroun		1688	20.6
Gebremariam et al., [47]	2016	Ethiopia	19–93	142	12.0
Toure et al., [48]	2016	Senegal		144	37.5
Kuate-Tegueu, et al., [49]	2016	Cameroun		120	26.7
Nakibuuka et al., [50]	2016	Uganda		254	32.7
Nakibuuka et al., [51]	2015	Uganda	19–99	127	18.1
Sarfo et al., [52]	2015	Ghana		12233	41.1
Nkoke et al., [53]	2015	Cameroun	62±13	254	23.2
Mamushet et al., [54]	2015	Ethiopia	18–90	71	23.9
Greffie et al., [55]	2015	Ethiopia	18–76	98	13.3
Deresse, et al., [56]	2015	Ethiopia	16–90	163	14.7
Sanya et al., [57]	2015	Nigeria	60.47 ± 13.60	302	21.2
Ekeh, et al., [58]	2015	Nigeria	<40–70+	120	33.3
Oduor, et al., [59]	2015	Kenya		155	27.7
Sarfo et al., [60]	2014	Ghana		265	43.4
Kwarisima, et al., [61]	2014	Uganda		128	43.8
Eze et al., [62]	2013	Nigeria	40–95	108	14.8
Femi et al., [63]	2013	Nigeria	18–90	273	37.0
Abubakar et al., [64]	2013	Nigeria		260	38.5
Okokhere et al., [65]	2013	Nigeria	66.22 ± 12.67	99	45.5
Atadzhanov et al., [66]	2012	Zambia	>18	250	40.4
Heikinheim et al., [67]	2012	Malawi	54.2 ±16.9	147	22.4
Agyemang et al., [68]	2012	Ghana	>24	1050	43.4
Watila et al., [69]	2012	Nigeria	56.4 ± 13.0	524	17.7
Ukoha et al., [70]	2012	Nigeria	30–79	35	20.0
Alkali, et al., [71]	2012	Nigeria	56.4 ± 12.7	272	18.8
Ossou-Nguiet, et al., [72]	2012	Congo Brazzaville		80	25.0
Owolabi et al., [73]	2012	Nigeria		273	37.4
Desalu, et al., [74]	2011	Nigeria	38–95	101	23.8
Owolabi et al., [75]	2011	Nigeria	18–40	71	23.9
Damasceno et al., [76]	2010	Mozambique	25–75+	651	33.3
Onwuchewa et al., [77]	2009	Nigeria	25–115	202	33.2
Wahab et al., [78]	2008	Nigeria	16–81	100	28.0
Longo-Mbenza et al., [79]	2008	Dr. Congo		212	44.3
Jowi, et al., [80]	2008	Kenya		80	5.0
Ndouongo et al., [81]	2007	Gabon	35–84	105	9.5
Garbusinski et al., [82]	2005	Gambia	24–96	162	39.5
Sagui et al., [83]	2005	Senegal		107	23.4
Ogun, et al., [84]	2005	Nigeria	30–120	708	40.0
Njoku, et al., [85]	2004	Nigeria		93	37.6
Walker, et al., [86]	2003	Gambia	20–93	106	26.4
Matuja et al., [87]	2023	Tanzania	64.5 ± 14.7	135	37.0
Wubshet et al., [88]	2023	Ethiopia		153	17.0
Jørgensen et al., [89]	2023	Tanzania		720	38.2
Neshuku et al., [90]	2023	Namibia		220	26.4
Youkee et al., [91]	2023	Sierra Leone		986	36.9
Ayehu et al., [92]	2022	Ethiopia	61 ± 12.85	554	27.1
Baye et al., [93]	2020	Ethiopia	18–100	448	12.5
Asres et al., [94]	2020	Ethiopia		170	20.0
Russell et al., [95]	2020	Sierra Leone	29–88	178	34.8
Asgedom et al., [96]	2020	Ethiopia		216	22.2
Abubakar et al., [97]	2017	Nigeria	55.51 ± 15.7	94	13.8
Admas et al., [98]	2022	Ethiopia	57.65±14.3	382	12.8
Adoukonou et al., [99]	2018	Benin	52 ± 15	85	27.1
Chongolo et al., [100]	2023	Tanzania		242	29.8
Gomes et al., [101]	2013	Mozambique		651	42.4
Kassie et al., [102]	2019	Ethiopia		232	29.7
Komolafe, et al., [103]	2007	Nigeria		135	15.6
Lekoubou et al., [104]	2016	Cameroun	57–72	1667	20.4
Mapoure, et al., [105]	2014	Cameroun		325	26.8
Matuja et al., [106]	2020	Tanzania		369	58.3
Danesi et al., [107]	2011	Nigeria		135	15.6
Nkusi et al., [108]	2017	Rwanda		96	20.8
Nutakki et al., [109]	2022	Zambia	60 ±18	324	23.8
Obiako et al., [110]	2011	Nigeria		66	57.6
Ojini, et al., [111]	2008	Nigeria		715	40.1
Olum et al., [112]	2021	Uganda	62.5±17.4	108	36.1
Prust et al., [113]	2021	Zambia	60 ± 16	125	29.6
Sarfo et al., [114]	2023	Ghana/Nigeria		3739	21.8
Walelgn et al., [115]	2021	Ethiopia		368	18.5

### 30-day in-hospital stroke case fatality

Table 2 shows the pooled 30-day in-hospital stroke case fatality rate, and the results of the subgroup analysis.

**Table 2:** Overall and sub-regional estimates of 30-day in-hospital stroke case fatality in Sub-Saharan Africa.

	Number of studies	Participants	Case fatality [CI:95%]	Heterogeneity (I^2^)
Overall estimate	93	42057	26.9% [24.5%-29.3%]	96.81
Sub-regional				
East Africa	42	10711	24% [21%-28%]	95.93
West Africa	43	29751	29% [25%-32%]	97.19
Central Africa	6	925	31% [20%-43%]	93.73
Southern Africa	2	670	25% [21%-28%]	0.01

The overall pooled estimates showed 27% [25%-29%] case fatality rate, but results were heterogeneity between studies [I^2^ = 97, p<0.000].

The pooled estimate for six studies conducted in Central Africa was the highest (31% [20%-43%]) case fatality but results were heterogenous between studies (I^2^ = 94%). The pooled estimate for forty-three studies conducted in West Africa was the next highest (29% [25%-32%]) case fatality, but results were also heterogenous between studies (I^2^ = 97%). This was followed by the pooled estimate for two studies conducted in Southern Africa which was 25% [21%-28%] and homogenous (I^2^ = 0%). The pooled estimate for forty-two studies conducted in East Africa was the lowest (24% [21%-28%]) but results were heterogenous between studies (I^2^ = 96%).

### Risk factors for 30-day in-hospital stroke case fatality

The risk factors associated with stroke case fatality are presented in Table 3. A total of ten (10) significant risk factors were identified. These were low Glasgow Coma Score <8 [24,28,34,35,48,50,58,61,63,73,95,96,100,105], high (≥10) National Institute Health Stroke Scale score [40,57,58,60,78,82,87,92,114], aspiration pneumonia [27,63,73,82,87,88,90,95,100,114], hemorrhagic stroke [29,40,92,95,105], brain edema/ intra-cranial pressure [28,34,88,114], hyperglycemia [35,40,50], untyped stroke (stroke diagnosis not confirmed by neuroimaging) [28,34,95], recurrent stroke [29,95], fever [27,82], and high creatinine levels [33].

**Table 3:** Risk factors associated with 30-day in-hospital stroke case fatality in Sub-Saharan Africa.

Authors	Low GCS Score	High NHISS score	Aspiration Pneumonia	Hemorrhagic Stroke	Untyped stroke	Hyper glycemia	High creatinine level	Fever	Recurrent stroke	Brain edema/ICP
Namale et al., [24]	√									
Dabilgou, et al., [27]			√					√		
Fekadu et al., [28]	√				√					√
Kamabu, et al., [29]				√					√	
Gebreyohannes, et al., [33]							√			
Fekadu et al., [34]	√				√					√
Tshituta et al., [35]	√					√				
Labodi et al., [40]		√		√		√				
Toure et al., [48]	√									
Nakibuuka et al. [50]	√					√				
Sanya et al., [57]		√	√							
Ekeh, et al., [58]	√	√								
Sarfo et al., [60]		√								
Kwarisima et al., [61]	√									
Femi et al., [63]	√		√							
Owolabi et al., [73]	√		√							
Wahab et al., [78]		√								
Garbusinski et al., [82]		√	√					√		
Matuja et al., [87]		√	√							
Wubshet et al., [88]			√							√
Neshuku et al., [90]			√							√
Ayehu et al., [92]		√		√						
Russel et al., [95]	√		√	√	√				√	
Asgedom et al., [96]	√									
Chongolo et al., [100]	√		√							
Mapoure et al., [105]	√			√						
Sarfo et al., [114]		√	√							√

GCS = Glasgow Coma Scale; NIHSS = National Institute of Health Stroke Scale; ICP = Intra-cranial Pressure.

### Risk of bias and quality assessment

Table 4 shows the quality of the studies and risk of bias. Seventy-six studies [11,24–26,28–31,34–37,39–48,50,52,53,55–61,63–69,71–76,78–93,96,98–104,106–108,110,111,113,114] showed low risk of bias and seventeen studies [27,33,38,37,49, 51,54,62,70,77,94,95,97,105, 109,112,115] showed moderate risk of bias. Twenty-two studies [26,29,32,34,39,41,46,48,50,60,61,67,68,75,76,83,85,86,92,98,110,111] had the highest overall score. One study Ukoha et al., [70] had the lowest overall score.

**Fig 2:**
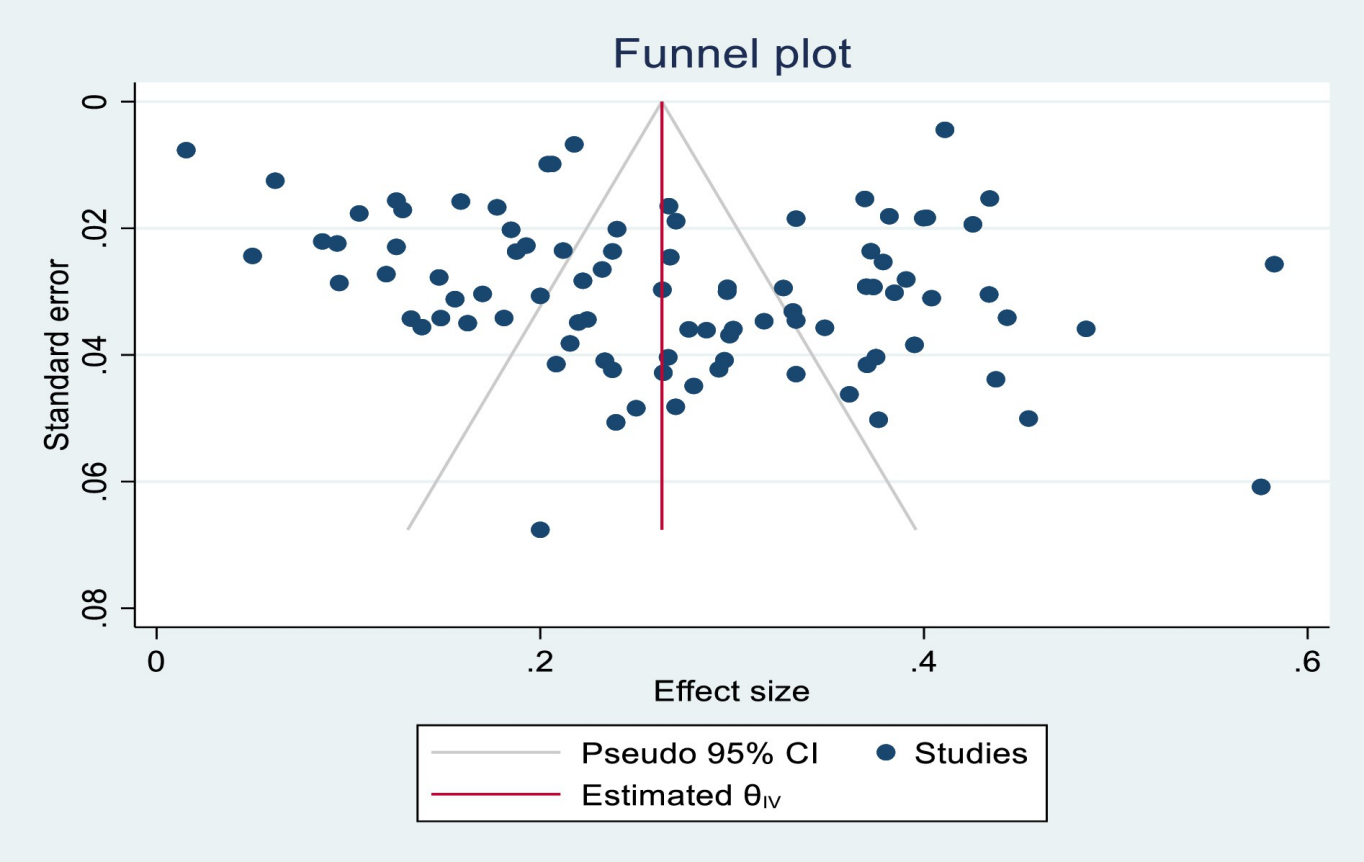
Funnel plot of publication bias.

Fig 2 shows the publication bias. Inspection of the funnel plot [Fig 2] showed no publication bias.

**Table 4:** Classification on methodological quality and risk of bias of included studies.

Study	Representative population	Representative Sampling frame	Random selection	Non-response bias	Data quality	Case definition	Study tool reliability and validity	Data collection consistency	Prevalence duration	Numerator/ Denominator suitability	Quality rating (0–10)	Overall risk of study bias
Desalu, et al., (74)	Low	Low	Low	Low	High	Low	High	High	Low	Low	7	Low
Fekadu, et al., (34)	Low	Low	Low	Low	Low	Low	Low	High	High	Low	8	Low
Alemayehu, et al., [11]	High	Low	Low	High	Low	Low	High	Low	Low	Low	7	Low
Namale et al., [24]	Low	Low	Low	Low	Low	Low	High	High	High	Low	7	Low
Mulugeta et al., [25]	High	Low	Low	Low	Low	Low	High	Low	Low	High	7	Low
Gadisa, et al., [26]	Low	Low	Low	Low	Low	Low	High	High	Low	Low	8	Low
Dabilgou, et al., [27]	Low	Low	Low	Low	Low	High	High	High	High	Low	6	Moderate
Fekadu, et al., [28]	Low	Low	Low	Low	Low	Low	High	High	High	Low	7	Low
Kamabu, et al., [29]	Low	Low	Low	Low	Low	Low	Low	High	High	Low	8	Low
Lisk, et al., [30]	High	Low	Low	Low	Low	Low	High	Low	Low	High	7	Low
Adoukonou et al., [31]	Low	Low	Low	Low	Low	Low	High	High	High	Low	7	Low
Kayode-Iyasere et al., [32]	Low	Low	Low	Low	Low	Low	High	High	Low	Low	8	Low
Gebreyohannes, et al., [33]	Low	Low	Low	Low	Low	High	High	High	High	Low	6	Moderate
Tshituta et al., [35]	Low	Low	Low	High	Low	Low	Low	High	High	Low	7	Low
Erkabu, et al., [36]	Low	Low	Low	Low	Low	High	High	Low	High	Low	7	Low
Asefa, et al., [37]	Low	Low	Low	High	Low	Low	High	High	High	Low	6	Moderate
Kaduka, et al., [38]	Low	Low	Low	Low	Low	High	High	High	High	Low	6	Moderate
Mbelesso et al., [39]	Low	Low	Low	Low	Low	Low	High	High	Low	Low	8	Low
Labodi et al., [40]	Low	Low	Low	Low	Low	Low	High	High	High	Low	7	Low
Gedefa et al., [41]	Low	Low	Low	Low	Low	Low	Low	High	High	Low	8	Low
Okeng’o et al., [42]	High	Low	Low	Low	Low	Low	High	Low	Low	High	7	Low
Kaseke et al., [43]	Low	Low	Low	Low	Low	Low	High	High	High	Low	7	Low
Sultan et al., [44]	Low	Low	Low	Low	Low	Low	High	High	High	Low	7	Low
Owolabi et al., [45]	Low	Low	Low	Low	Low	Low	High	High	Low	High	7	Low
Lekoubou, et al., [46]	Low	Low	Low	Low	Low	Low	High	High	Low	Low	8	Low
Gebremariam et al., [47]	Low	Low	Low	Low	Low	High	High	Low	High	Low	7	Low
Toure et al., [48]	Low	Low	Low	Low	Low	Low	High	High	Low	Low	8	Low
Kuate-Tegueu, et al., [49]	Low	Low	Low	Low	Low	High	High	High	High	Low	6	Moderate
Nakibuuka et al., [50]	Low	Low	Low	Low	Low	Low	Low	High	High	Low	8	Low
Nakibuuka et al., [51]	Low	Low	Low	Low	Low	High	High	High	High	Low	6	Moderate
Sarfo et al., [52]	High	Low	Low	Low	Low	Low	High	Low	Low	High	7	Low
Nkoke et al., [53]	High	Low	Low	Low	Low	Low	High	Low	Low	High	7	Low
Mamushet et al., [54]	Low	Low	Low	Low	Low	High	High	High	High	Low	6	Moderate
Greffie et al., [55]	Low	Low	Low	Low	Low	Low	High	High	Low	High	7	Low
Deresse, et al., [56]	Low	Low	Low	Low	Low	Low	High	High	High	Low	7	Low
Sanya et al., [57]	Low	Low	Low	Low	Low	Low	High	High	High	Low	7	Low
Ekeh, et al., [58]	High	Low	High	Low	Low	Low	High	Low	Low	Low	7	Low
Oduor, et al., [59]	Low	Low	Low	Low	Low	Low	High	High	High	Low	7	Low
Sarfo et al., [60]	Low	Low	Low	Low	Low	Low	High	High	Low	Low	8	Low
Kwarisima, et al., [61]	Low	Low	Low	Low	Low	Low	Low	High	High	Low	8	Low
Eze et al., [62]	Low	Low	Low	Low	Low	High	High	High	High	Low	6	Moderate
Femi et al., [63]	Low	Low	Low	Low	Low	Low	High	High	Low	High	7	Low
Abubakar et al., [64]	Low	Low	Low	High	Low	Low	High	Low	High	Low	7	Low
Okokhere et al., [65]	Low	Low	Low	Low	Low	High	High	Low	High	Low	7	Low
Atadzhanov et al., [66]	Low	Low	Low	High	Low	Low	High	High	Low	Low	7	Low
Heikinheim et al., [67]	Low	Low	Low	Low	Low	Low	High	High	Low	Low	8	Low
Agyemang et al., [68]	Low	Low	Low	Low	Low	Low	High	High	Low	Low	8	Low
Watila et al., [69]	Low	Low	Low	Low	Low	Low	High	High	High	Low	7	Low
Ukoha et al., [70]	High	Low	Low	High	Low	Low	High	Low	High	High	5	Moderate
Alkali, et al., [71]	Low	Low	Low	High	Low	Low	High	Low	High	Low	7	Low
Ossou-Nguiet, et al., [72]	Low	Low	Low	Low	Low	Low	High	High	High	Low	7	Low
Owolabi et al., [73]	High	Low	Low	Low	Low	Low	High	Low	Low	High	7	Low
Owolabi et al., [75]	Low	Low	Low	Low	Low	Low	High	High	Low	Low	8	Low
Damasceno et al., [76]	Low	Low	Low	Low	Low	Low	Low	High	High	Low	8	Low
Onwuchewa et al., [77]	Low	Low	High	Low	High	Low	Low	High	High	Low	6	Moderate
Wahab et al., [78]	Low	Low	Low	Low	Low	High	High	Low	High	Low	7	Low
Longo-Mbenza et al., [79]	Low	Low	Low	Low	Low	High	High	Low	High	Low	7	Low
Jowi, et al., [80]	Low	Low	Low	Low	Low	High	High	Low	High	Low	7	Low
Ndouongo, et al., [81]	Low	Low	Low	Low	Low	High	High	Low	High	Low	7	Low
Garbusinski et al., [82]	High	Low	Low	Low	Low	Low	High	Low	Low	High	7	Low
Sagui et al., [83]	Low	Low	Low	Low	Low	Low	Low	High	High	Low	8	Low
Ogun, et al., [84]	Low	Low	Low	Low	Low	Low	High	High	Low	High	7	Low
Njoku, et al., [85]	Low	Low	Low	Low	Low	Low	High	High	Low	Low	8	Low
Walker, et al., [86]	Low	Low	Low	Low	Low	Low	Low	High	High	Low	8	Low
Matuja et al., [87]	Low	Low	Low	Low	Low	Low	High	High	High	Low	7	Low
Wubshet et al., [88]	Low	High	Low	Low	Low	Low	High	High	Low	Low	7	Low
Jørgensen et al., [89]	High	Low	Low	Low	Low	Low	High	Low	Low	High	7	Low
Neshuku et al., [90]	Low	Low	Low	Low	Low	Low	High	High	Low	High	7	Low
Youkee et al., [91]	High	Low	Low	Low	Low	Low	High	Low	Low	High	7	Low
Ayehu et al., [92]	Low	Low	Low	Low	Low	Low	High	High	Low	Low	8	Low
Baye et al., [93]	High	Low	Low	High	Low	Low	High	Low	Low	Low	7	Low
Asres et al., [94]	Low	Low	High	Low	Low	Low	High	High	High	Low	6	Moderate
Russell et al., [95]	Low	Low	Low	Low	Low	High	High	High	High	Low	6	Moderate
Asgedom et al., [96]	Low	High	Low	Low	Low	Low	Low	High	High	Low	7	Low
Abubakar et al., [97]	Low	Low	Low	High	Low	Low	High	High	High	Low	6	Moderate
Admas et al., [98]	Low	Low	Low	Low	Low	Low	Low	High	High	Low	8	Low
Adoukonou et al., [99]	Low	Low	Low	Low	High	Low	High	High	Low	Low	7	Low
Chongolo et al., [100]	Low	Low	Low	Low	Low	High	High	Low	High	Low	7	Low
Gomes et al., [101]	Low	Low	Low	Low	Low	Low	High	High	High	Low	7	Low
Kassie et al., [102]	Low	Low	Low	Low	Low	Low	High	High	Low	High	7	Low
Komolafe, et al., [103]	High	Low	Low	Low	Low	Low	High	Low	Low	High	7	Low
Lekoubou et al., [104]	Low	Low	Low	Low	Low	Low	High	High	Low	High	7	Low
Mapoure, et al., [105]	Low	Low	Low	High	Low	High	Low	High	High	Low	6	Moderate
Matuja et al., [106]	Low	Low	Low	Low	Low	Low	High	High	Low	High	7	Low
Danesi et al., [107]	Low	Low	Low	Low	Low	High	High	Low	High	Low	7	Low
Nkusi et al., [108]	Low	Low	Low	Low	Low	High	High	Low	High	Low	7	Low
Nutakki et al., [109]	Low	Low	Low	Low	Low	High	High	High	High	Low	6	Moderate
Obiako et al., [110]	Low	Low	Low	Low	Low	Low	Low	High	High	Low	8	Low
Ojini, et al., [111]	Low	Low	Low	Low	Low	Low	High	High	Low	Low	8	Low
Olum et al., [112]	Low	Low	Low	Low	Low	High	High	High	High	Low	6	Moderate
Prust et al., [113]	Low	Low	Low	Low	Low	High	High	Low	High	Low	7	Low
Sarfo et al., [114]	Low	Low	Low	Low	Low	Low	High	High	Low	High	7	Low
Walelgn et al., [115]	Low	Low	Low	Low	Low	High	High	High	High	Low	6	Moderate

## Discussion

The primary aim of this systematic review and meta-analysis was to examine 30-day in-hospital stroke case fatality and associated risk factors in sub-Saharan Africa. A total of 93 hospital-based retrospective and prospective cohort studies, involving 42,057 persons with stroke across 19 countries in sub-Saharan Africa were included in the review. The meta-analysis of pooled data revealed 27% 30-day in-hospital stroke case fatality rate. Comparatively, this estimate is slightly higher than the pooled estimate of 22% reported in a previous systematic review and meta-analysis [12]. A possible explanation for this disparity could be the larger number of studies (i.e., 93 studies) included in the present review, as compared to the previous one (i.e., 27 studies), further highlighting the need for this current review. The 27% case fatality found in the present review is somewhat higher than the trends in high income countries [116]. Possible explanations may be the rising incidence of stroke in low- and middle-income countries such as those in SSA, and the limited availability of resources for acute medical and rehabilitation care for persons with stroke in many SSA countries [16,117,118].

Sub-group analysis revealed varying rates of 30-day in-hospital stroke case fatality across the SSA regions, with the highest rates found in Central Africa (31%), followed by West Africa (29%), Southern Africa (25%), and East Africa (24%). The observed variance in case fatality rates across sub-regions could be partly explained by the gross domestic product and infrastructural development of each collective sub-region, rather than their individual countries. People’s conceptualization of disease and health and their health-seeking behaviors are partly determined by their culture, socioeconomic development of society, and availability of health facilities and services [119]. The high case fatality and the variance observed across the sub-regions found in this current study, may thus be a consequence of sociocultural-economic factors. Delayed hospital visits, admissions and obsolete diagnostic equipment can lead to misdiagnosis and mismanagement, resulting in increased case fatality rates.

An important finding of this review was the risk factors associated with 30-day stroke case fatality in SSA. The identified risk factors (i.e., stroke severity, stroke type, untyped stroke, and post-stroke complications) are similar to findings of previous systematic reviews conducted within the sub-region [12,120]. The identified risk for stroke case fatality may be linked to clinical and socioeconomic factors. Hemorrhagic strokes tend to be more severe compared to ischemic strokes and are linked to a significant rise in mortality during the first three months of a stroke. Persons with stroke in SSA are likely to receive an untyped stroke diagnosis due to limited resources including but not limited to financial constraints to afford a requisite diagnostic test such as a Computed Tomography (CT) scan, and lack of available diagnostic facilities and services, increasing susceptibility for misdiagnosis and mismanagement. Many countries in SSA have limited healthcare personnel, frequent CT scan malfunctions, and high costs associated with medical imaging, all of which could contribute to late or failure to confirm the stroke diagnosis [28,34,95], which hampers management. The likely phenomenon of untyped stroke coupled with inability to afford required diagnostic test and lack of diagnostic services likely contributes to the high case fatality in SSA [26,67,93].

Given the relatively high stroke case fatality in SSA, there is a need for effective policies and interventions to minimize the incidence and mortality from stroke at the national, regional, and local levels across countries in SSA. Context-specific health policies to encourage the routine assessment of vulnerable population groups for early detection of risk factors for case fatality is warranted. Additionally, there is the need to enhance public health education and raise awareness about the risk factors for stroke among residents in the region. Improving the healthcare in most SSA countries is essential to addressing in-hospital stroke case fatality. There is thus the need to equip health facilities with the requisite personnel, diagnostic equipment, and procedures to help improve stroke care and rehabilitation within SSA. Such interventions will not only enhance the quality of care provided but also reduce recovery time, hospital stays, and stroke-related fatalities.

### Strengths and limitations

This review possesses a number of strengths. The adoption of a well-established systematic review and meta-analysis methodologies, which are aligned with internationally recognized standards and recommendations, coupled with the large number of included studies is a strength of this review.

The review also has some limitations. The studies included in this review exhibited considerable heterogeneity. Readers should therefore be cautious when interpreting the pooled estimates. Emphasis should instead be placed on the distribution in each category and the observed patterns in the data [121]. Additionally, the inclusion of only studies published in English, likely underestimate the extent of the case fatality in the sub-region as studies may have been published in other languages.

## Conclusion

The present review examined the current scientific evidence regarding 30-day in-hospital stroke case fatality and associated risk factors in sub-Saharan Africa and found 27% stroke case fatality rate. This suggests that at least one in every four hospitalized stroke patients dies within 30 days in SSA. However, most of the identified risk factors for the case fatality were modifiable. This highlights the need for context-tailored health policies, clinical guidelines, and treatment protocols to help with early detection and prevention of risk factors to minimize in-hospital stroke mortality in SSA.

## Supporting information

S1 Checklist PRISMA 2020 checklist. (DOCX)

S1 Data Data associated with manuscript. (XLSX)

S1 Table Search strategy. (DOCX)
