# Correction: 30-day in-hospital stroke case fatality and significant risk factors in sub-Saharan–Africa: A systematic review and meta-analysis

**DOI:** 10.1371/journal.pgph.0006115

**Published:** 2026-03-11

**Authors:** Martin Ackah, Louise Ameyaw, Richard Appiah, David Owiredu, Hosea Boakye, Webster Donaldy, Cosmos Yarfi, Ulric S. Abonie

The seventh author’s name is spelled incorrectly. The correct name is: Cosmos Yarfi.
