# Supplementary material for: 30-day in-hospital stroke case fatality and significant risk factors in sub-Saharan–Africa: A systematic review and meta-analysis
Source: PLOS Glob Public Health. 2024 Jan 19;4(1):e0002769. doi: 10.1371/journal.pgph.0002769 (PMC10798456; doi:10.1371/journal.pgph.0002769)
Supplement: S1 Table — (DOCX) [file pgph.0002769.s003.docx]

**PUBMED=11,703**

|  | Search term |
| --- | --- |
|  | ‘’Cerebrovascular accident’’ OR Stroke OR ‘’stroke outcome*’’ |
|  | ‘’In-hospital’’ OR ‘’in-patient*’’ OR outcome* |
|  | Mortality OR ‘’30- day mortality’’ OR death OR ‘’case-fatality’’ OR ‘’30-case-fatality’’ |
|  | risk factor* OR associated factor* |
|  | sub- Saharan Africa OR Angola OR Benin OR Botswana OR Burkina Faso OR Burundi OR Cameroon OR Cape Verde OR Central African Republic OR Chad OR Comoros OR Congo OR Cote d'Ivoire OR Djibouti OR Equatorial Guinea OR Ethiopia OR Gabon OR The Gambia OR Ghana OR Guinea OR Guinea- Bissau OR Kenya OR Lesotho OR Liberia OR Madagascar OR Malawi OR Mali OR Mauritania OR Mauritius OR Mozambique OR Namibia OR Niger OR Nigeria OR Rwanda OR Sao Tome and Principe OR Senegal OR Seychelles OR Sierra Leone OR Somalia OR South Africa OR Sudan OR Swaziland OR Tanzania OR Togo OR Uganda OR Zaire OR Zambia OR Zimbabwe |
|  | #1 AND #2 AND #3 AND #4 AND #5 |
|  | Limit to 1990- September,2023 |

**CINAHL ultimate=1,499**

( ‘’Cerebrovascular accident’’ OR Stroke OR ‘’stroke outcome*’’ OR ‘’In-hospital’’ OR ‘’in-patient*’’ OR outcome* ) AND ( Mortality OR ‘’30- day mortality’’ OR death OR ‘’case-fatality’’ OR ‘’30-case-fatality’’ ) AND ( risk factor* OR associated factor* ) AND ( sub- Saharan Africa OR Angola OR Zimbabwe OR Botswana OR Burkina Faso OR Burundi OR Cameroon OR Cape Verde OR Central African Republic OR Chad OR Comoros OR Congo OR Cote d'Ivoire OR Djibouti OR Equatorial Guinea OR Ethiopia OR Gabon OR The Gambia OR Ghana OR Guinea OR Guinea- Bissau OR Kenya OR Lesotho OR Liberia OR Madagascar OR Malawi OR Mali OR Mauritania OR Mauritius OR Mozambique OR Namibia OR Niger OR Nigeria OR Rwanda OR Sao Tome and Principe OR Senegal OR Seychelles OR Sierra Leone OR Somalia OR South Africa OR Sudan OR Swaziland OR Tanzania OR Togo OR Uganda OR Zaire OR Zambia OR Zimbabwe )

**APA PyscNet=354 [PsycINFO=349 and PyschArticleS=5]**

**354**Results for **Any Field**: (‘’Cerebrovascular accident’’ OR Stroke OR ‘’stroke outcome*’’ OR ‘’In-hospital’’ OR ‘’in-patient*’’ OR outcome*) AND (Mortality OR ‘’30- day mortality’’ OR death OR ‘’case-fatality’’ OR ‘’30-case-fatality’’) AND (risk factor* OR associated factor*) AND (sub- Saharan Africa OR Angol OR Zimbabwe OR Botswana OR Burkina Faso OR Burundi OR Cameroon OR Cape Verde OR Central African Republic OR Chad OR Comoros OR Congo OR Cote d'Ivoire OR Djibouti OR Equatorial Guinea OR Ethiopia OR Gabon OR The Gambia OR Ghana OR Guinea OR Guinea- Bissau OR Kenya OR Lesotho OR Liberia OR Madagascar OR Malawi OR Mali OR Mauritania OR Mauritius OR Mozambique OR Namibia OR Niger OR Nigeria OR Rwanda OR Sao Tome and Principe OR Senegal OR Seychelles OR Sierra Leone OR Somalia OR South Africa OR Sudan OR Swaziland OR Tanzania OR Togo OR Uganda OR Zaire OR Zambia OR Zimbabwe)

**Africa Journal Online=58**

’Cerebrovascular accident’’ OR Stroke OR ‘’stroke outcome*’’ OR ‘’In-hospital’’ OR ‘’in-patient*’’ OR outcome* ) AND ( Mortality OR ‘’30- day mortality’’ OR death OR ‘’case-fatality’’ OR ‘’30-case-fatality’’ ) AND ( risk factor* OR associated factor* ) AND ( sub- Saharan Africa OR Angola OR Zimbabwe OR Botswana OR Burkina Faso OR Burundi OR Cameroon OR Cape Verde OR Central African Republic OR Chad OR Comoros OR Congo OR Cote d'Ivoire OR Djibouti OR Equatorial Guinea OR Ethiopia OR Gabon OR The Gambia OR Ghana OR Guinea OR Guinea- Bissau OR Kenya OR Lesotho OR Liberia OR Madagascar OR Malawi OR Mali OR Mauritania OR Mauritius OR Mozambique OR Namibia OR Niger OR Nigeria OR Rwanda OR Sao Tome and Principe OR Senegal OR Seychelles OR Sierra Leone OR Somalia OR South Africa OR Sudan OR Swaziland OR Tanzania OR Togo OR Uganda OR Zaire OR Zambia OR Zimbabwe

**Google scholar=61**

Stroke mortality in sub-Saharan Africa
